# Survival Outcome of Partial Cystectomy versus Transurethral Bladder Tumor Resection in T1 High-Grade Bladder Cancer Patients: A Propensity Score Matching Study

**DOI:** 10.1155/2022/3016725

**Published:** 2022-10-25

**Authors:** Zheng Ping, Xiangpeng Zhan, Tao Chen, Yunwei Zheng, Ming Jiang, Yu Li, Bin Fu

**Affiliations:** ^1^Department of Urology, Shangrao Municipal Hospital, Shangrao, Jiangxi, China; ^2^Department of Urology, The First Affiliated Hospital of Nanchang University, Nanchang, Jiangxi, China

## Abstract

**Purpose:**

Partial cystectomy was investigated as a method of bladder preservation with better disease outcomes than transurethral bladder tumor resection in T1 high-grade bladder cancer patients. *Method and materials*. The national Surveillance, Epidemiology, and End Results database (SEER) (2004–2015) were used to obtain patients diagnosed with T1 high-grade bladder cancer, and finally, 25263 patients were enrolled in our study. The Kaplan–Meier method with the log-rank test was performed to analyze the outcome of overall survival (OS) and cancer-specific survival (CSS) between patients undergoing partial cystectomy (PC), transurethral resection of bladder tumor (TURBT), or radical cystectomy (RC). Moreover, the propensity score matching (PSM) and multivariable Cox proportional hazard model were also utilized in the study.

**Results:**

Ultimately, 24635 patients were undergoing TURBT, while 190 and 438 patients were, respectively, assigned to the PC and RC groups. Compared with patients with TURBT, a tendency of a higher proportion of higher older and male patients was observed in the PC group. When matching with RC patients, patients in the PC group were commonly older and had bigger tumor sizes and single tumors (All *P* < 0.05). After 1 : 1 PSM, 190 patients with TURBT and 160 patients receiving PC were selected. In survival analysis, the patients in the PC group had a higher survival probability of both OS and CSS before and after PSM compared with those in the TURBT group. Meanwhile, no significant differences were observed between the RC and PC groups in OS and CSS analysis. Moreover, multivariable Cox regression showed that PC was a protective factor for overall mortality (ACM) and cancer-specific mortality (CSM) compared with TURBT in T1 high-grade patients (All *P* < 0.05).

**Conclusion:**

Patients undergoing partial cystectomy were shown to have a better outcome compared with those with transurethral bladder tumor resection in T1 high-grade bladder cancer patients. Partial cystectomy could be the more worthwhile choice for bladder preservation in T1 high-grade bladder cancer patients.

## 1. Introduction

Urothelial bladder cancer (UBC) is the 9th most common cancer globally and the most common cancer in the urinary system [1, 2]. 75% of patients diagnosed with UBC are nonmuscle-invasive bladder cancer (NMIBC), and 20% of NMIBC patients are T1 high-grade (T1HG) [3, 4].

T1 high-grade tumors are invasive and have a high probability of disease recurrence and progression to muscle invasion compared with other NMIBC tumors [5]. Now, there are still challenges to the treatment of T1 high-grade tumors, and the main difficulties are choosing between bladder preservation like transurethral resection of the bladder (TURB) with maintenance bacillus Calmette-Guerin (BCG) and aggressive treatment such as radical cystectomy [2, 6]. The bladder preservation approach was considered the ideal treatment and used widely but had a high risk of disease progression and recurrence. For example, a retrospective study that enrolled 1155 patients with T1 high-grade bladder tumors suggested about 25% of cases after the first TURBT presented residual high-grade disease [7]. Meanwhile, in patients with primary T1HG/G3 treated with maintenance BCG, the residual T1HG/G3 tumor after TURBT would lead to a worse prognosis [8]. In addition, some evidence proved that patients with older age or carcinoma in situ might not be effective in BCG therapy [9, 10]. The difference in the efficacy of BCG for different patients was proved to associate with baseline basophil count, routine systemic inflammatory markers, and systemic combined inflammatory score [11–13]. Radical cystectomy was confirmed as the best chance at cure while it might be overtreatment for some patients with T1HG tumors [5, 6, 14]. These results suggested that the existing treatment for patients with T1HG bladder cancer might still not be satisfactory. Urologists are committed to finding an ideal bladder preservation method with better oncology outcomes given the negative effects of radical cystectomy on patients' quality of life.

Over the past decade, partial cystectomy as an approach to bladder preservation was explored as a viable alternative to radical cystectomy for some specifically selected MIBC patients, and partial cystectomy offered acceptable outcomes and adequate local control [15, 16]. Partial cystectomy as an alternative to TURBT and RC might gain a balance between disease prognosis and patient's quality of life for T1HG bladder cancer patients. However, partial cystectomy was not utilized frequently, ranging from 7 to 10% of all cystectomies based on the studies of the National Cancer Database [17]. To our knowledge, no studies focused on PC's effect in T1HG bladder cancer patients due to insufficient cases. To better understand the treatment effect of PC compared with TURBT and RC in T1HG patients, we searched the Surveillance, Epidemiology, and End Results (SEER) database (2004–2015). We enrolled 25264 patients with T1HG tumors who had received surgery of PC, TURBT, or RC. Ultimately, the purpose of this study is to present a relatively good level of evidence for urologists and patients to choose the most suitable and ideal treatment for T1HG bladder cancer patients.

## 2. Materials and Methods

### 2.1. Patient Database and Study Population

All patient data were obtained from the Surveillance, Epidemiology, and End Results (SEER), which represented the demographic composition and cancer incidence of the United States population. The conditions for inclusion were as follows: (1) Year of diagnosis: 2004–2015; (2) T stage: T1 high-grade (CS site-Specific Factors 1 code 20); (3) Histology behavior: transitional cell carcinoma (TCC) and papillary transitional cell carcinoma (PTCC); (4) Surgical approach using surgery codes: transurethral bladder tumor resection, partial cystectomy, and radical cystectomy. The following exclusion criteria were also applied: (1) N1, N2, N3, NX (485); (2) M1, MX (270); (3) Grade unknown (1263); (4) Tumor size unknown (7453); (5) Marital status unknown (417).

### 2.2. Definition of Variables

In our study, patients with T1 high-grade bladder cancer were divided into three groups according to the “RX Summ-Surg Prim Site (1998+)” column in the SEER database, which contained TURBT, PC, and RC. Demographic characteristics of patients included age of diagnosis, sex, race, and marital status. Cancer characteristics incorporated tumor location, tumor grade, histology of the tumor, tumor size, and the number of tumors. Treatment of patients contained surgical approaches, radiation recode, and chemotherapy recode. The race was divided into three categories: white, black, and other styles (American/Indian/Alaska/Native/Asian/Pacific Islander). Marital status was classified into three types: married, SDW (separated, divorced, and widowed), and single. Tumor grade was separated into three groups, including grade I or grade II, III, and IV, to facilitate analysis. Tumor size and the number of tumors were converted to categorical variables as continuous variables. Other variables included: (1) Radiation recode (no/unknown, yes); (2) Histology: transitional cell carcinoma (TCC) and papillary transitional cell carcinoma (PTCC); (3) chemotherapy recode (no/unknown, yes). (4) Tumor site (trigone, dome, lateral, anterior, posterior, bladder neck, ureteric orifice, other sites including overlapping lesion of bladder and bladder, NOS).

### 2.3. Endpoints

The all-cause mortality (ACM) and cancer-specific mortality (CSM) were the primary endpoints in our study. All-cause mortality matched with any cause of death, and cancer-specific mortality referred to only those who died of bladder cancer. Any patients still were coded as alive if they died after the study cutoff date. The unit of survival time was recorded as a month.

### 2.4. Statistical Analyses

Propensity score matching (PSM) was performed by SPSS version 22.0 (IBM Corp, Armonk, NY). Baseline characteristics of patients receiving PC were assessed to confirm whether significant differences were compared with patients in other groups. *Two-samplet-tests* were used for continuous variables while the *Chi-square test* was for categorical variables to verify heterogeneity between groups. The *P* < 0.50 was recognized as significant, and all *P* values of results were two-tailed. The mean ± SD, medians, and interquartile ranges were all presented for continuous variables while frequencies and their proportions were for categorical variables. Survival curves of overall survival (OS) and cancer-specific survival (CSS) were constructed by R studio using the Kaplan–Meier method with the log-rank test. Meanwhile, subgroup analysis of OS and CSS was performed in T1HG patients stratified by age (<70 years and >70 years), sex (male and female), histology (transitional cell carcinoma and papillary transitional cell carcinoma), tumor size (<3 cm and >3 cm), number of tumors (single and multiple), and grade (Grade III and Grade IV), which could further study the outcomes of T1HG patients. In addition, univariate and multivariate Cox regression analysis was also applied to analyze the prognosis of PC. All results of Cox regression analysis were presented with hazard ratios (HR) and 95% confidence intervals (95% CI). The multivariate Cox regression model adjusted confounding factors which contained age, sex, race, marital status, grade, histology, tumor size, number of tumors, tumor sites, radiotherapy, and chemotherapy. To balance baseline characteristics of the study population (including age, sex, race, marital status, grade, histology, radiotherapy, chemotherapy, tumor size, tumor site, and number of tumors), propensity score matching (PSM) was performed by SPSS. For PSM, patients receiving TURBT, or PC were matched 1 : 1 with a caliper set at 0.001 on the based sample size, but a caliper of 0.02 was applied for patients receiving PC or RC.

## 3. Results

### 3.1. Demographics and Clinical Characteristics of the Study Population

Finally, 25263 patients were enrolled in our study. There were 24635 patients receiving TURBT, while 190 and 438 patients were, respectively, assigned to the PC and RC groups. The median follow-up time was 43 months. The number of death cases was 12077, 69, and 214 for those undergoing TURBT, PC, and RC, respectively. The all-cause mortality rates were 49.02%, 36.32%, and 32.67% for those undergoing TURBT, PC, and RC, respectively, while the cancer-specific mortality rates were 26.13%, 19.47%, and 18.93%. Tables 1 and 2 revealed the baseline characteristics of the study population before and after PSM. As Table 1 showed, patients in the TURBT groups were older than those in the PC group (72.9 ± 10.371 vs. 71.5 ± 9.830 *P*=0.014). Meanwhile, a higher percentage of males was shown in PC patients compared with TURBT patients. Similarly, the PC group had a higher rate of dome, anterior, and posterior tumors. However, there were still statistical differences between the TURBT and PC groups on histology, tumor size, tumor size, and the number of tumors (all *P* < 0.05). When matching with RC patients, patients in the PC group were older (71.5 ± 9.830 vs. 67.9 ± 9.625, *P* < 0.001). Moreover, bigger tumor sizes and single tumors were more common in the PC group (all *P* < 0.05). After PSM, significant differences existed in tumor size and tumor site between PC and RC patients.

### 3.2. Survival Analyses and Subgroup Analysis

**Figure 1:**
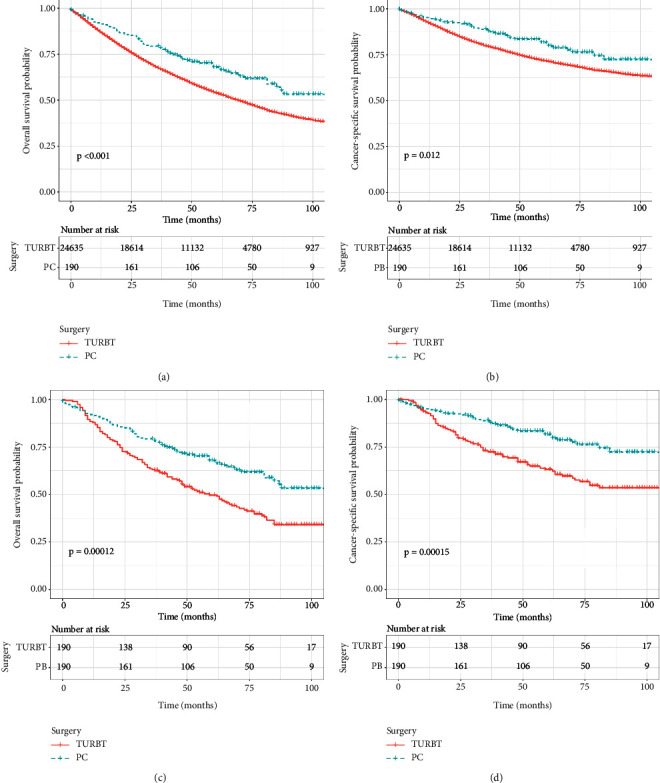
Survival curve with the Kaplan–Meier method between TURBT and PC patients: (a) before PSM for OS; (b) before PSM for CSS (c) after PSM for OS (d) after PSM for CSS.

**Figure 2:**
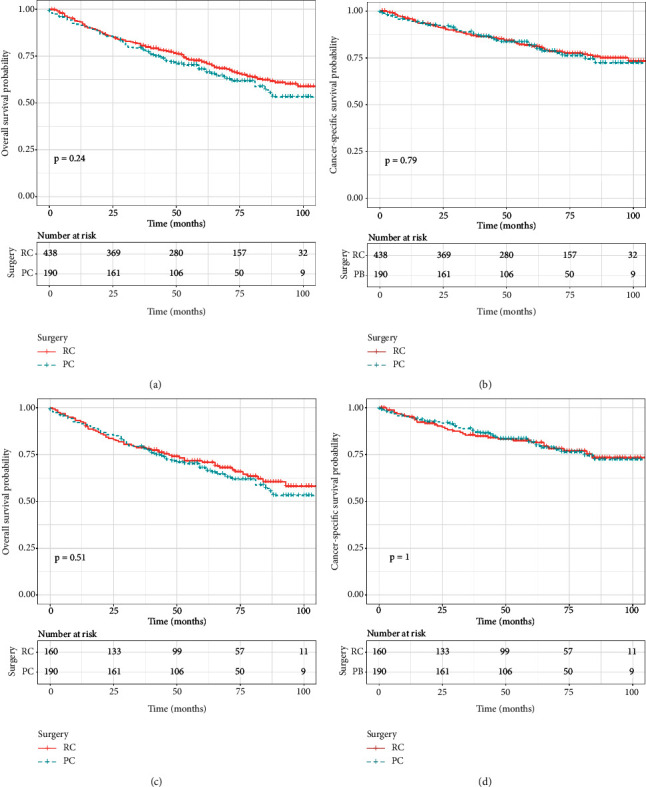
Survival curve with the Kaplan–Meier method between RC and PC patients: (a) before PSM for OS; (b) before PSM for CSS (c) after PSM for OS (d) after PSM for CSS.

**Figure 3:**
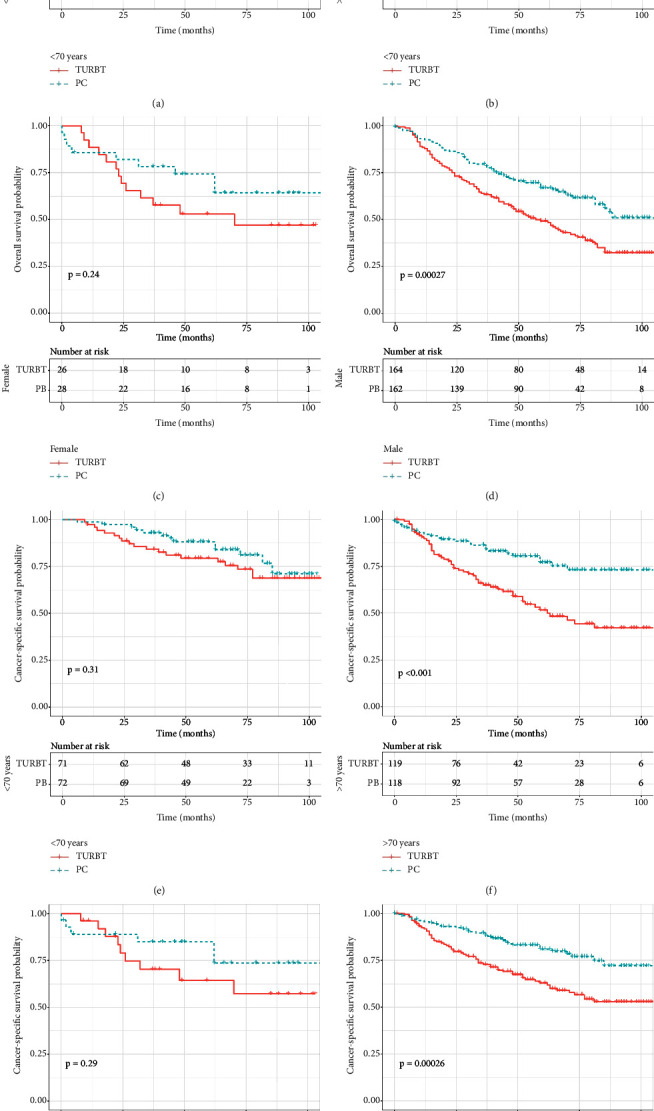
Survival curve with the Kaplan–Meier method between TURBT and PC patients after PSM subgroup analysis by age and gender: (a–d) for OS; (e–h) for CSS.

**Figure 4:**
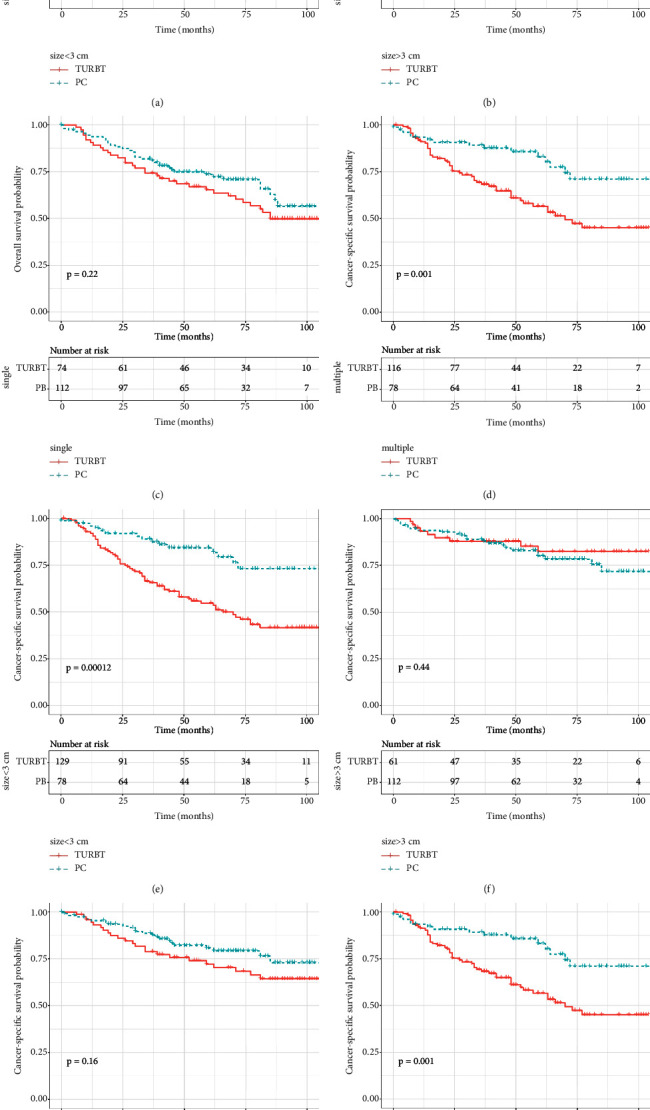
Survival curve with the Kaplan–Meier method between TURBT and PC patients after PSM subgroup analysis by size and number of tumour: (a–d) for OS; (e–h) for CSS.

**Figure 5:**
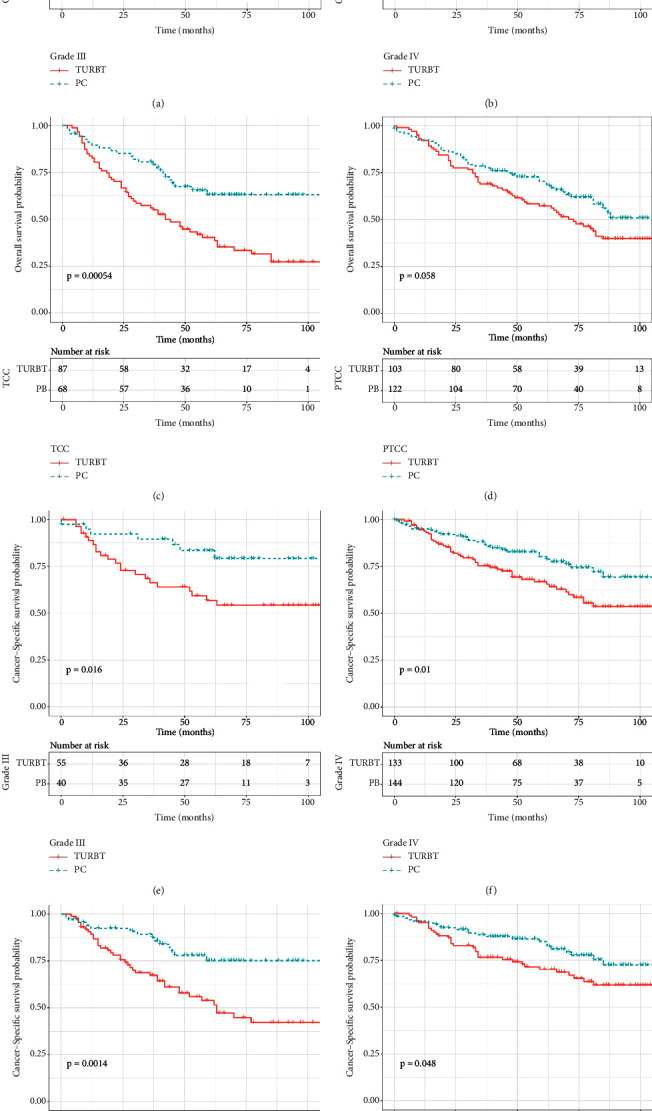
Survival curve with the Kaplan–Meier method between TURBT and PC patients after PSM subgroup analysis by grade and histology: (a–d) for OS; (e–h) for CSS.

**Figure 6:**
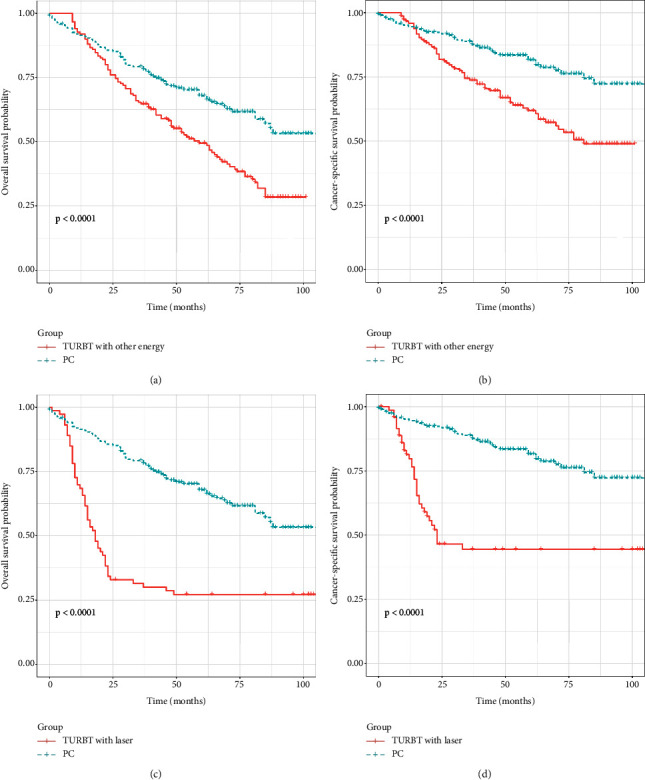
Survival curve with the Kaplan–Meier method between TURBT and PC patients after PSM subgroup analysis: (a and b) (TURBT with other energy); (c and d) (TURBT with lasers).

**Figure 7:**
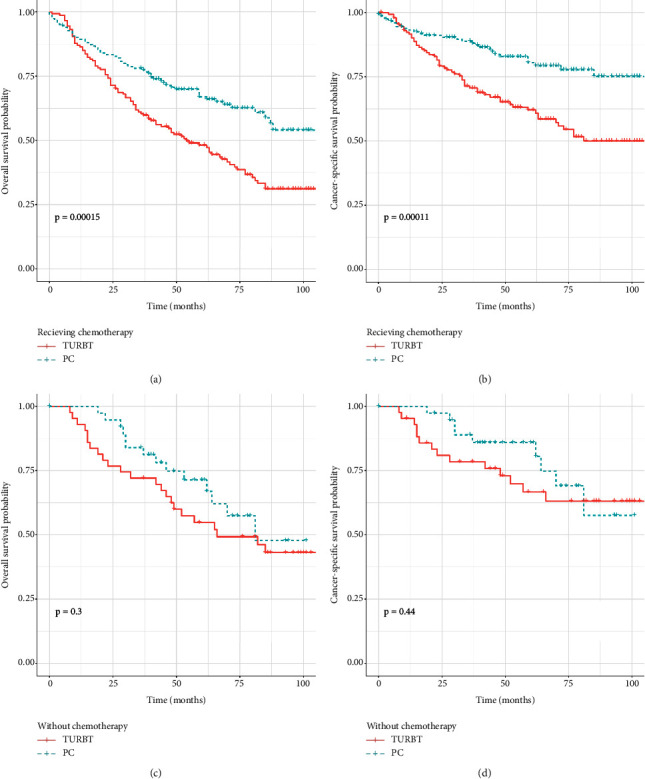
Figure 6 survival curve with the Kaplan–Meier method between TURBT and PC patients after PSM subgroup analysis by whether receiving chemotherapy: (a and c) for OS; (b and d) for CSS.

As survival curves showed, patients receiving PC had a better prognosis of OS and CSS compared with those with TURBT before and after PSM (Figures 1(a)–1(d), all *P* < 0.05). In addition, no significant differences in OS and CSS were observed in terms of surgery of PC and RC before and after PSM (Figures 2(a)–2(d); all *P* > 0.05). When stratified by age, we found that patients over 70 years could obtain survival benefits of OS and CSS from PC while this effect was not observed in patients younger than 70 years (Figures 3(b) and 3(f), *P* < 0.001; Figures 3(a) and 3(e), *P* > 0.05). Moreover, the subgroup stratified by gender to validate the different survival outcomes between PC and TURBT revealed that a better survival probability of OS and CSS was obtained in the male patients (Figures 3(d) and 3(h)). At the same time, similar conditions did not appear in female patients which might account for a small sample size (Figures 3(c) and 3(g)). We also conducted subgroup analysis stratified by tumor size and a number of tumors, and we obtained the results that patients with tumor size bigger than 3 cm or multiple tumors could gain a better prognosis from PC matching with TURBT in terms of OS and CSS (Figure 4). Patients in the PC group all showed longer CSS and OS than those in the TURBT group when stratified by grade and histology, while the statistical difference failed to show in terms of OS in PTCC patients (Figures 5(a)–5(h)). Compared with patients undergoing TURBT with lasers or other energy, patients with PC all presented a better OS and CSS (all *P* < 0.001) (Figure 6). For patients receiving chemotherapy, a better OS and CSS were observed among patients undergoing PC when compared to those with TURBT (Figures 7(a) and 7(b)). However, we failed to obtain statistical differences in OS and CSS in patients without chemotherapy (Figures 7(c) and 7(d)).

### 3.3. Multivariable Analysis of PC for CSM and ACM before and after PSM

Table 3 demonstrated multivariable Cox proportional hazard models. After adjustments for confounding factors like age, sex, race, marital status, grade, histology, radiotherapy, chemotherapy, tumor size, tumor site, and the number of tumors, the adjusted model all presented that PC was a protective factor for all-cause mortality (ACM) (PC vs. TURBT; HR = 0.717, 95% CI = 0.565–0.908, *P*=0.006), while this result failed to gain in term of cancer-specific mortality (CSM) (PC vs TURBT; HR = 0.756, 95% CI = 0.547–1.044, *P*=0.09) before PSM. However, after 1 : 1 PSM, statistical differences on ACM (PC vs TURBT; HR = 0.667, 95% CI = 0.467–0.952, *P*=0.026) and CSM (PC vs TURBT; HR = 0.589, 95% CI = 0.370–0.939, *P*=0.026) were both witnessed.

## 4. Discussion

Our study aimed to investigate an approach in T1HG patients, which could be a better choice than TURBT to preserve the bladder and retained not bad disease prognosis compared with RC. In this study, we confirmed the results that the T1HG patients could gain survival benefit of OS and CSS from PC when matched with TURBT, and most subgroup analyses confirmed the result. Meanwhile, statistical differences were failed to observe between PC and RC on CSS and OS analysis. Moreover, compared to TURBT, multivariate cox regression analysis revealed that PC was a protective factor of ACM and CSM in T1HG patients.

Formerly, partial cystectomy has experienced a resurgence. A growing body of literature has argued that PC might be a viable alternative to RC for select muscle-invasive bladder cancer (MIBC) patients [15, 16, 18]. Considering potential complications of RC like severe blood loss, infections, paralytic ileus, and issues with wound healing and negative effect on long-term life, urologist and patients transferred their focus on PC, which could preserve a complete bladder and function of voiding, avoids urinary diversion, and maintain sexual function [18]. In addition, *Umberto Capitanio's* study based on the SEER database got similar results, which showed PC did not enervate the prognosis of OS and CSS in selected patients [19]. A subsequent retrospective study has also issued a similar conclusion that no differences were seen between PC and RC in metastasis-free or cancer-specific survival [15, 20, 21]. However, some critics of partial cystectomy proclaimed that PC was an incomplete cancer operation following a significant risk of recurrence for MIBC patients and delaying the best treatment time [18, 22]. T1HG tumor was aggressive cancer but in the early stages compared to MIBC tumors. Therefore, partial cystectomy might be worth trying as a method of bladder preservation to replace RC.

As far as we know, few studies were focusing on the impact of TURBT and PC. However, there was research revealed that 50% of patients diagnosed with T1 bladder cancer in the first TURBT displayed residual tumors when a second TURBT was applied, while 10–25% of those patients were confirmed as muscle-invasive bladder cancer [22–25]. TURBT showed a lousy power of cancer control and accurate pathology report based on the results of these data. Meanwhile, PC removed local tumors, which deleted full-thickness bladder containing lesions, while the alarm of intraoperative bladder perforation still existed in surgery of TURBT. Furthermore, more pathological data obtained like muscle infiltration and lymph node metastasis was the superiority of PC [16, 18, 20]. Simultaneously, BCG shortage was also a challenge for traditional treatment like TURBT following BCG [26]. In summary, PC was a bladder preservation strategy with significant quantity advantages superior to TURBT, and it was an option worth considering for T1HG patients.

In this study, compared with TURBT patients, patients receiving PC tended to be young (71.5 ± 9.830 vs 72.9 ± 10.371, *P*=0.014) and had a higher proportion of male (85.3% vs 79.2%, *P*=0.041). When matched with the RC group, the PC group appeared older (71.5 ± 9.830 vs 67.9 ± 9.625, *P* < 0.001) and were more likely to have a single tumor (58.9% vs 41.8%, *P* < 0.001). This result confirmed with criteria of adopting PC developed by *MD Anderson* [16, 20]. However, a higher proportion of bigger tumors size was observed in the PC group, which might be the cause of insufficient sample size.

Survival analysis in propensity score-matched subgroups was performed to precisely compare the efficacy between PC and TURBT. In the subgroup of age >70, male, tumour size<3 cm, multiple, grade III, and IV, histology of transitional cell carcinoma and papillary transitional cell carcinoma, T1HG patients with PC all showed better survival outcomes of OS and CSS than those with TURBT. Nevertheless, it failed to gain significant differences in OS and CSS analysis in the subgroup of age <70, female, tumour size>3 cm, and single. More similar studies were needed to confirm these results.

Up to our knowledge, it is the first study to propose the idea of performing PC as a method of bladder preservation in T1HG patients. Utilization of propensity matching score method to perform potential confounding factors and reduce selective bias was also the advantage of this study. Detailed subgroup analysis also made the results more convincing. However, there were still some limitations in our study. Firstly, unavoidable selection bias still existed in this retrospective study even PSM was applied. Then, the number of patients receiving PC is still not enough to go further study, and it might be the reason for insignificant differences in subgroup analysis and multivariate cox regression analysis. In addition, the SEER database lacked some important disease outcomes such as tumor recurrence and progression, and they are also essential evaluation indexes for surgery. Meanwhile, more detail information of partial cystectomy like surgical approach was failed to obtain due to limitation of SEER database. Ultimately, statistical differences in demographics and clinical characteristics of the study population remained after PSM. The patients of the RC and PC group failed to match 1 : 1 in performing PSM owing to insufficient patients with RC (160 for RC vs 190 for PC after PSM). Consequently, A prospective study with a larger sample size and a more rigorous design is needed to verify these results.

Finally, some problems of PC were needed to attach importance to. For some special patients with carcinoma in situ (CIS), tricky tumor location including ureteral orifice or bladder neck and multiple tumors, we need to carefully consider the effect of partial cystectomy for these patients [16, 18, 20]. In addition, the COVID-19 pandemic posed unprecedented challenges to our health system and delays in treatment schedule and disease management were proposed [27]. Therefore, for T1HG bladder cancer, which was considered to be a chronic and expensive disease, the management might face greater challenges in the future. More appropriate combination therapy and follow-up strategies should be explored.

## 5. Conclusions

Partial cystectomy was proven to have a better outcome of overall survival and cancer-specific survival when compared with transurethral bladder tumor resection in T1 high-grade patients. In addition, no difference in overall survival and cancer-specific survival was observed between patients undergoing partial cystectomy and those undergoing radical cystectomy. Partial cystectomy could be a worthwhile choice for bladder preservation in T1 high-grade bladder cancer patients.

## Figures and Tables

**Table 1:** Clinicopathological features between TURBT and PC before and after propensity score matching.

Variables	No PSM			PSM		
	TURBT (*n* = 24635)	PC (*n* = 190)	*P* value	TURBT (*n* = 190)	PC (*n* = 190)	*P* value
*Age (year)*						
Mean ± SD	72.9 ± 10.371	71.5 ± 9.830	0.014^∗^	72.7 ± 11.448	71.5 ± 9.830	0.260
Median (25th–75th percentile)	72 (67.5–82)	72 (67.5–77.5)	0.016^∗^	77.5 (62–82)	72 (67.5–77.5)	0.068
Sex			0.041^∗^			0.769
Female	5115 (20.8%)	28 (14.7%)		26 (13.7%)	28 (14.7%)	
Male	19520 (79.2%)	162 (85.3%)		164 (86.3%)	162 (85.3%)	
Race			0.572			0.072
White	21859 (88.7%)	171 (90.0%)		162 (85.3%)	171 (90.0%)	
Black	1448 (5.9%)	12 (6.3%)		10 (5.3%)	12 (6.3%)	
Other style	1328 (5.4%)	7 (3.7%)		18 (9.5%)	7 (3.7%)	
Marital status			0.055			0.859
Married	15420 (62.6%)	132 (69.5%)		135 (71.1%)	132 (69.5%)	
SDW	6285 (25.5%)	34 (17.9%)		30 (15.8%)	34 (17.9%)	
Single	2930 (11.9%)	24 (12.6%)		25 (13.2%)	24 (12.6%)	
Grade			0.544			0.090
Grade I or Grade II	517 (2.1%)	6 (3.2%)		2 (1.1%)	6( 3.2%)	
Grade III	4926 (20.0%)	40 (21.1%)		55 (28.9%)	40 (21.1%)	
Grade IV	19192 (77.9%)	144 (75.8%)		133 (70.0%)	144 (75.8%)	
Histology			0.119			0.047^∗^
TCC	7527 (30.6%)	68 (35.8%)		87 (45.8%)	68 (35.8%)	
PTCC	17108 (69.4%)	122 (64.2%)		103 (54.2%)	122 (64.2%)	
Radiotherapy			0.535			0.2
No/unknown	24081 (97.8%)	187 (98.4%)		183 (96.3%)	187 (98.4%)	
Yes	554 (2.2%)	3 (1.6%)		7 (3.7%)	3 (1.6%)	
Chemotherapy			0.127			0.618
No/unknown	18389 (74.6%)	151 (79.5%)		147 (77.4%)	151 (79.5%)	
Yes	6246 (25.4%)	39 (20.5%)		43 (22.6%)	39 (20.5%)	
Tumor size			0.267			<0.001^∗^
≤3 cm	10919 (44.3%)	78 (41.1%)		129 (67.9%)	78 (41.1%)	
3–6 cm	9833 (39.9%)	74 (38.9%)		54 (28.4%)	74 (38.9%)	
>6 cm	3883 (15.8%)	38 (20.0%)		7 (3.7%)	38 (20.0%)	
Tumor site			<0.001^∗^			<0.001^∗^
Trigone	1266 (5.1%)	1 (0.5%)		19 (10.0%)	1 (0.5%)	
Dome	1235 (5.0%)	29 (15.3%)		11 (5.8%)	29 (15.3%)	
Lateral	4941 (20.1%)	30 (15.8%)		35 (18.4%)	30 (15.8%)	
Anterior	704 (2.9%)	10 (5.3%)		8 (4.2%)	10 (5.3%)	
Posterior	2453 (10.0%)	22 (11.6%)		23 (12.1%)	22 (11.6%)	
Bladder neck	784 (3.2%)	2 (1.1%)		7 (3.7%)	2 (1.1%)	
Ureteric orifice	584 (2.4%)	4 (2.1%)		4 (2.1%)	4 (2.1%)	
Other sites	12668 (51.4%)	92 (48.4%)		83 (43.7%)	92 (48.4%)	
Number of tumors			0.880			<0.001^∗^
Single	14655 (59.5%)	112 (58.9%)		74 (38.9%)	112 (58.9%)	
Multiple	9980 (40.5%)	78 (41.1%)		116 (61.1%)	78 (41.1%)	

*Survival time (month)*						
Mean	47.498	55.121	<0.001^∗^	51.763	55.121	0.015^∗^
Median (25th–75th percentile)	55 (37–77)	55.5 (37–77)	<0.001^∗^	48 (23–81.25)	55.5 (37–77)	0.236

PC: partial cystectomy; TURBT: transurethral bladder tumor resection; other race: American/Indian/Alaska/Native/Asian/Pacific Islande; SDW: separated, divorced or widowed; other sites: overlapping lesion of bladder, bladder, NOS; TCC: transitional cell carcinoma, PTCC: papillary transitional cell carcinoma. PSM, propensity score matching. ^∗^Statistically significant.

**Table 2:** Clinicopathological features between RC and PC before and after propensity score matching.

Variables		No PSM			PSM		
		RC (*n* = 438)	PC (*n* = 190)	*P* value	RC (*n* = 160)	PC (*n* = 190)	*P* value
*Age (year)*							
Mean ± SD	67.9 ± 9.625		71.5 ± 9.830	<0.001^∗^	69.9 ± 9.408	71.5 ± 9.830	0.121
Median (25th–75th percentile)	67.5 (62–72)		72 (66–77.5)	<0.001^∗^	72 (62–77.5)	72 (66–77.5)	0.063
Sex				0.968			0.817
Female	64 (14.6%)		28 (14.7%)		25 (15.6%)	28 (14.7%)	
Male	374 (85.4%)		162 (85.3%)		135 (84.4%)	162 (85.3%)	
Race				0.496			0.318
White	387 (88.4%)		171 (90.0%)		144 (90.0%)	171 (90.0%)	
Black	25 (5.7%)		12 (6.3%)		6 (3.8%)	12 (6.3%)	
Other style	26 (5.9%)		7 (3.7%)		10 (6.3%)	7 (3.7%)	
Marital status				0.826			0.393
Married	301 (68.7%)		132 (69.5%)		117 (73.1%)	132 (69.5%)	
SDW	74 (16.9%)		34 (17.9%)		30 (18.8%)	34 (17.9%)	
Single	63 (14.4%)		24 (12.6%)		13 (8.1%)	24 (12.6%)	
Grade				0.703			0.215
Grade I or Grade II	9 (2.1%)		6 (3.2%)		1 (0.6%)	6 (3.2%)	
Grade III	95 (21.7%)		40 (21.1%)		38 (23.8%)	40 (21.1%)	
Grade IV	334 (76.3%)		144 (75.8%)		121 (75.6%)	144 (75.8%)	
Histology				0.13			0.602
TCC	185 (42.2%)		68 (35.8%)		53 (33.1%)	68 (35.8%)	
PTCC	253 (57.8%)		122 (64.2%)		107 (66.9%)	122 (64.2%)	
Radiotherapy				0.653			0.403
No/unknown	433 (98.9%)		187 (98.4%)		159 (99.4%)	187 (98.4%)	
Yes	5 (1.1%)		3 (1.6%)		1 (0.6%)	3 (1.6%)	
Chemotherapy				0.842			0.868
No/unknown	345 (78.8%)		151 (79.5%)		126 (78.8)	151 (79.5%)	
Yes	93 (21.2%)		39 (20.5%)		34 (21.3%)	39 (20.5%)	
Tumor size				<0.001^∗^			0.042^∗^
≤3 cm	323(73.7%)		78 (41.1%)		87 (54.4%)	78 (41.1%)	
3–6 cm	86(19.6%)		74 (38.9%)		46 (28.7%)	74 (38.9%)	
>6 cm	29 (6.6%)		38 (20.0%)		27 (16.9%)	38 (20.0%)	
Tumor site				<0.001^∗^			0.041^∗^
Trigone	14 (3.2%)		1 (0.5%)		8 (5.0%)	1 (0.5%)	
Dome	17 (3.9%)		29 (15%)		15 (9.4%)	29 (15.3%)	
Lateral	49 (11.2%)		30 (15.8%)		34 (21.3%)	30 (15.8%)	
Anterior	11 (2.5%)		10 (5.3%)		7 (4.4%)	10 (5.3%)	
Posterior	29 (6.6%)		22 (11.6%)		12 (7.5%)	22 (11.6%)	
Bladder neck	11 (2.5%)		2 (1.1%)		4 (2.5%)	2 (1.1%)	
Ureteric orifice	6 (1.4%)		4 (2.1%)		1 (0.6%)	4 (2.1%)	
Other sites	301 (68.7%)		92 (48.4%)		79 (49.4%)	92 (48.4%)	
Number of tumor				<0.001^∗^			0.457
Single	183 (41.8%)		112 (58.9%)		88 (55.0%)	112 (58.9%)	
Multiple	255 (58.2%)		78 (41.1%)		72 (45.0%)	78 (41.1%)	

*Survival time (month)*							
Mean	60.021		55.121	0.40	59.106	55.121	0.196
Median (25th–75th percentile)	62 (39–86)		55.5 (37–77)	0.038^∗^	60.5 (39–82.75)	55.5 (37–77)	0.157

PC: partial cystectomy; RC: radical cystectomy; other race: American/Indian/Alaska/native/Asian/Pacific Islande; SDW: separated, divorced or widowed; other sites: overlapping lesion of bladder, bladder, NOS; TCC: transitional cell carcinoma, PTCC: papillary transitional cell carcinoma. PSM, propensity score matching. ^∗^Statistically significant.

**Table 3:** Univariate and multivariable Cox proportional hazard model for PC.

Outcomes	PC HR (95% CI), (TURBT ref.)	*P* value
*Overall mortality*		
Nonadjusted	0.650 (0.513–0.824)	*P* < 0.001^*∗*^
Adjusted	0.717 (0.565–0.908)	*P*=0.006^*∗*^
PSM nonadjusted	0.562 (0.417–0.758)	*P* < 0.001^*∗*^
PSM adjusted	0.667 (0.467–0.952)	*P*=0.026^*∗*^

*Cancer-specific mortality*		
Nonadjusted	0.664 (0.481–0.917)	0.013^∗^
Adjusted	0.756 (0.547–1.044)	*P*=0.09
PSM nonadjusted	0.474 (0.319–0.705)	*P* < 0.001^*∗*^
PSM adjusted	0.589 (0.370–0.939)	*P*=0.026^*∗*^

Nonadjusted: univariate Cox regression analysis for PC. All model adjusted for: age, sex, race, marital status, grade, histology, radiotherapy, chemotherapy, tumor size, tumor site, number of tumors. PC: partial cystectomy; PSM: propensity score matching. ^∗^Statistically significant.

## Data Availability

The data in this article come from the SEER database This data can be found here: https://seer.cancer.gov/data/.
